# Influenza B Viruses with Mutation in the Neuraminidase Active Site, North Carolina, USA, 2010–11

**DOI:** 10.3201/eid1711.110787

**Published:** 2011-11

**Authors:** Katrina Sleeman, Tiffany G. Sheu, Zack Moore, Susan Kilpatrick, Shikha Garg, Alicia M. Fry, Larisa V. Gubareva

**Affiliations:** Centers for Disease Control and Prevention, Atlanta, Georgia, USA (K. Sleeman, T.G. Sheu, S. Garg, A.M. Fry, L.V. Gubareva); Battelle, Atlanta (T.G. Sheu); North Carolina Department of Health and Human Services, Raleigh, North Carolina, USA (Z. Moore, S. Kilpatrick)

**Keywords:** zanamivir, oseltamivir, peramivir, neuraminidase inhibitor, viruses, North Carolina, influenza, influenza B, mutation, United States

## Abstract

Oseltamivir is 1 of 2 antiviral medications available for the treatment of influenza B virus infections. We describe and characterize a cluster of influenza B viruses circulating in North Carolina with a mutation in the neuraminidase active site that may reduce susceptibility to oseltamivir and the investigational drug peramivir but not to zanamivir.

Influenza B viruses are responsible for sporadic seasonal influenza illness and can be associated with severe illness and death. In the United States, there are 2 classes of antiviral drugs licensed by the Food and Drug Administration for treatment of influenza infections. The adamantanes are ineffective against influenza B viruses, which limits the available antiviral options to 2 neuraminidase inhibitors (NAIs), inhaled zanamivir and oral oseltamivir. Influenza B viruses seem to have reduced susceptibility to NAIs compared with influenza A viruses on the basis of neuraminidase inhibition (NAI) assays (*1**,**2*). Furthermore, in clinical studies, changes conferring either resistance or reduced susceptibility to NAIs have been identified in the neuraminidase (NA) of influenza B viruses isolated from patients after treatment (*3**–**6*). Although the use of an antiviral agent can lead to the development of drug resistance, influenza B viruses with a reduced NAI susceptibility have also been recovered from patients with no history of exposure (*5**,**7**–**10*). It is therefore plausible that such mutations may be naturally occurring within the NA of influenza B viruses.

## The Study

During routine influenza antiviral susceptibility surveillance, an influenza B virus, B/North Carolina/11/2010, with reduced susceptibility to oseltamivir and the investigational NAI peramivir was detected by using the fluorescent NAI assay based on IC_50_ values (amount of NAI required to inhibit 50% of viral NA activity). According to the current algorithm, viruses with elevated IC_50_ values, when compared with a drug-susceptible control reference virus, are further investigated by using either conventional sequencing or pyrosequencing. Sequence analysis for the NA gene of B/North Carolina/11/2010 showed a novel substitution, present as a mixed population, of isoleucine (I) to valine (V) at position 221 (B NA numbering corresponds to 222 in N2 NA amino acid numbering). A substitution of I to threonine (T) at 221 has previously been associated with reduced susceptibility to NAIs in influenza B viruses (*1**,**5**,**9*). Moreover, reduced susceptibility to oseltamivir has been reported in viruses with variation at the corresponding residue (223, N1 NA numbering) in the pandemic (H1N1) 2009 virus (*11**,**12*) and in influenza A/H5N1 (*13*) and A/H3N2 viruses (*14*).

Subsequent fluorescent NAI testing of isolates recovered during surveillance showed a cluster of 14 influenza B viruses from North Carolina with elevated oseltamivir IC_50_ values compared with reference wild-type influenza B, wild-type pandemic (H1N1) 2009, and wild-type A(H3N2) viruses; a similar trend was observed for peramivir IC_50_ values (Table 1). When comparing the pandemic (H1N1) 2009 virus with the oseltamivir-resistance conferring H275Y substitution and an influenza A (H3N2) virus with the oseltamivir-resistance conferring E119V substitution, the North Carolina B viruses showed intermediate susceptibility (Table 1). The influenza B virus carrying the R152K substitution was resistant to all NAIs compared with the influenza B viruses with I221V (Table 1). In the chemiluminescent NAI assay, the oseltamivir IC_50_ values for the I221V variants were greater than that for the E119V influenza A (H3N2) virus variant, which has been associated with oseltamivir resistance (Table 1) (*15*). Pyrosequencing analysis showed I221V as well as wild-type (I221) in the propagated viruses used in the NAI assays. The presence of wild-type variants is likely to reduce IC_50_ values.

**Table 1:** Comparison of influenza virus susceptibility, North Carolina, USA, 2010−11 influenza seasons*

Type/subtype and strain designation	Virus subset	NA change	Fluorescent NAI assay, IC_50_ ± SD, nmol/L (-fold)				Chemiluminescent NAI assay, IC_50_ ± SD nmol/L (-fold)		
			Zanamivir	Oseltamivir	Peramivir		Zanamivir	Oseltamivir	Peramivir
Influenza B									
B/North Carolina/11/2010	Test	I221V/I	8.58 (3)	20.39 (6)	2.77 (8)		5 (2)	8.97 (5)	1.26 (5)
B/North Carolina/03/2011	Test	I221V/I	8.13 (3)	18.98 (6)	2.43 (7)		6.37 (2)	4.98 (3)	1.19 (4)
B/North Carolina/13/2010	Test	I221V/I; K360E	6.42 (2)	17.76 (5)	2.6 (7)		3.67 (1)	4.84 (3)	1.29 (5)
B/North Carolina/07/2011	Test	I221V/I; S283N	7.93 (3)	22.31 (7)	2.95 (8)		4.82 (2)	6.01 (3)	1.26 (5)
B/North Carolina/10/2011	Control	WT	4.34 (2)	10.67 (2)	0.64 (2)		2.82 (1)	2.72 (1)	0.41 (1)
2010–2011 influenza B, n = 39	Surveillance	WT	3.31 ± 0.91 (1)	8.33 ± 0.28 (3)	0.61 ± 0.18 (2)		2.48 ± 0.89 (1)	2.77 ± 0.79 (1)	0.38 ± 0.20 (1)
**B/Memphis/20/1996**	Reference	WT	2.59 ± 0.50 (1)	3.25 ± 0.9 (1)	0.35 ± 0.03 (1)		2.96 ± 0.64 (1)	1.88 ± 0.33 (1)	0.28 ± 0.04 (1)
B/Memphis/20/1996	Reference	R152K	66.14 ± 28.45 (26)	700.25 ± 61.06 (215)	269.78 ± 38.21 (771)		53.46 ± 11.59 (18)	177.20 ± 36.72 (94)	62.83 ± 38.02 (224)
Pandemic (H1N1) 2009									
**A/California/07/2009**	Reference	WT	0.26 ± 0.02 (1)	0.22 ± 0.08 (1)	0.08 ± 0.01 (1)		0.24 ± 0.04 (1)	0.21 ± 0.03 (1)	0.07 ± 0.01 (1)
A/Texas/48/2009	Reference	H275Y	0.37 ± 0.04 (1)	165.42 ± 24.15 (752)	16.32 ± 2.12 (204)		0.36 ± 0.06 (1)	58.77 ± 10.99 (280)	7.50 ± 1.30 (107)
Influenza A (H3N2)									
**A/Washington/01/2007**	Reference	WT	0.40 ± 0.07 (1)	0.12 ± 0.01 (1)	0.13 ± 0.01 (1)		0.79 ± 0.08 (1)	0.11 ± 0.02 (1)	0.13 ± 0.01 (1)
A/Texas/12/2007	Reference	E119V	0.40 ± 0.05 (1)	43.81 ± 1.90 (365)	0.16 ± 0.02 (1)		0.57 ± 0.09 (1)	3.37 ± 0.63 (31)	0.16 ± 0.03 (1)

*NA, neuraminidase; NAI, neuraminidase inhibition; IC_50_, 50% inhibitory concentration; WT, wild type. Mean IC_50_ values for wild-type reference control viruses for each type/subtype are in **boldface** and were used to calculate the -fold differences for their respective type/subtypes as indicated in parentheses. Test, influenza B viruses from North Carolina study carrying the I221V substitution. Control, influenza B virus with identical NA sequence as B/North Carolina/11/2010 with the exception of being wild-type (I) at position 221. Surveillance, influenza B viruses collected in the US during October 2010−December 2010 tested in routine influenza surveillance activities in both the fluorescent and chemiluminescent NAI assays. Reference, susceptible and resistant reference viruses used as controls in NAI assays. IC_50_ values for reference viruses represent the average taken from 5 replicates.

A total of 258 influenza B virus isolates from domestic and foreign laboratories submitted to the Centers for Disease Control and Prevention for routine surveillance were screened for the I221V substitution by using single-nucleotide polymorphism (SNP) pyrosequencing analysis (*10*). All viruses were wild type at this position, with the exception of the 14 viruses from North Carolina with reduced susceptibility in the NAI assay (Table 2). All 14 viruses were collected from patients in North Carolina during November 2010 through February 2011.

**Table 2:** Percentage composition of isoleucine and valine at position 221 in the neuraminidase of virus isolates and clinical specimens with reduced susceptibility to oseltamivir and peramivir, North Carolina, USA, 2010−11 influenza season*

Strain designation	Date of collection	Clinical specimen			Virus isolate	
		% Isoleucine	% Valine		% Isoleucine	% Valine
B/North Carolina/02/2010	2010 Nov 10	NA	NA		20	80
B/North Carolina/11/2010	2010 No 29	5	95		38	62
B/North Carolina/06/2010	2010 Dec 6	NA	NA		15	85
B/North Carolina/07/2010	2010 Dec 6	NA	NA		19	81
B/North Carolina/08/2010	2010 Dec 7	NA	NA		24	76
B/North Carolina/10/2010	2010 Dec 17	8	92		13	87
B/North Carolina/12/2010	2010 Dec 20	9	91		15	85
B/North Carolina/13/2010	2010 Dec 21	10	90		17	83
B/North Carolina/03/2011	2011 Jan 5	38	62		36	64
B/North Carolina/06/2011	2011 Jan 24	7	93		15	85
B/North Carolina/01/2011	2011 Feb 1	11	89		13	87
B/North Carolina/02/2011	2011 Feb 1	8	92		14	86
B/North Carolina/07/2011	2011 Jan 31	8	92		15	85
B/North Carolina/08/2011	2011 Feb 10	F	F		12	88
B/Memphis/20/1996	NA	NA	NA		100	0

*NA, not available; F, failed in pyrosequencing analysis. 10% is the standard cutoff value for the presence of a single-nucleotide polymorphism because of limitations of the pyrosequencing assay.

Because some susceptibility-altering NA mutations have been shown to arise from virus propagation in tissue culture (*15*), pyrosequencing analysis at position 221 in available matching clinical specimens was performed to rule out cell culture selection. The I221V substitution was identified in the 9 available matching clinical specimens (Table 2). Notably, most of the clinical specimens contained higher percentages of the V221 variant compared with the matching virus isolates, which may indicate a potential selective pressure for the wild-type variant (I221) in cell culture.

An epidemiologic investigation and enhanced surveillance was initiated in cooperation with the North Carolina Department of Health and Human Services. Of 220 patients with influenza B virus infections in North Carolina during November 2010 through March 2011, specimens from 209 patients underwent pyrosequencing analysis. Specimens from 45 (22%) patients from 13 counties contained the I221V mutation based on SNP pyrosequencing analysis; patient median age was 12 years (range 6 months–60 years). Among 199 patients with available antiviral treatment information, specifically for oseltamivir use, none had documented exposure to the virus before specimen collection. This finding may indicate that influenza B viruses carrying the I221V mutation are co-circulating with wild-type influenza B viruses in North Carolina.

## Conclusions

Although the NA change I221V has been seen among the N1 NA subtype of influenza A viruses (*1**,**13*), such a change has not been reported in influenza B viruses. Amino acid 221 is known to be a highly conserved residue of the NA enzyme active site. To date, all influenza B viruses with the I221V substitution appear to be limited geographically; however, monitoring is ongoing. Although oseltamivir IC_50_ values obtained with the influenza B viruses carrying the I221V substitution are similar to those seen with influenza A(H3N2) viruses carrying the oseltamivir-resistance conferring substitution E119V (Table 1), the clinical significance of the altered susceptibility associated with I221V in influenza B viruses is unknown at this time and warrants further investigation. Furthermore, such variant-dependent elevated IC_50_ values highlight the need for establishing a correlation between laboratory-determined IC_50_ values and clinical resistance.

Phylogenetic analysis of the hemagglutinin gene of the North Carolina B viruses carrying the I221V change in the NA is consistent with the B Victoria lineage (Figure, panel A). Similarly, phylogenetic analysis of the NA gene demonstrated that the North Carolina B viruses with the I221V change also belong to the B Victoria lineage and form a cluster because of the I221V substitution (Figure, panel B). As of March 2011, of the 438 influenza B viruses isolated in the United States, 94% were antigenically characterized as B/Brisbane/60/2008-like (B-Victoria lineage) (www.cdc.gov/flu/weekly). The cluster of North Carolina influenza B viruses carrying the I221V mutation antigenically matched the current influenza B component of the seasonal influenza vaccine. Data collected from an ongoing epidemiologic and clinical correlation study will be the subject of a more detailed future report.

**Figure:**
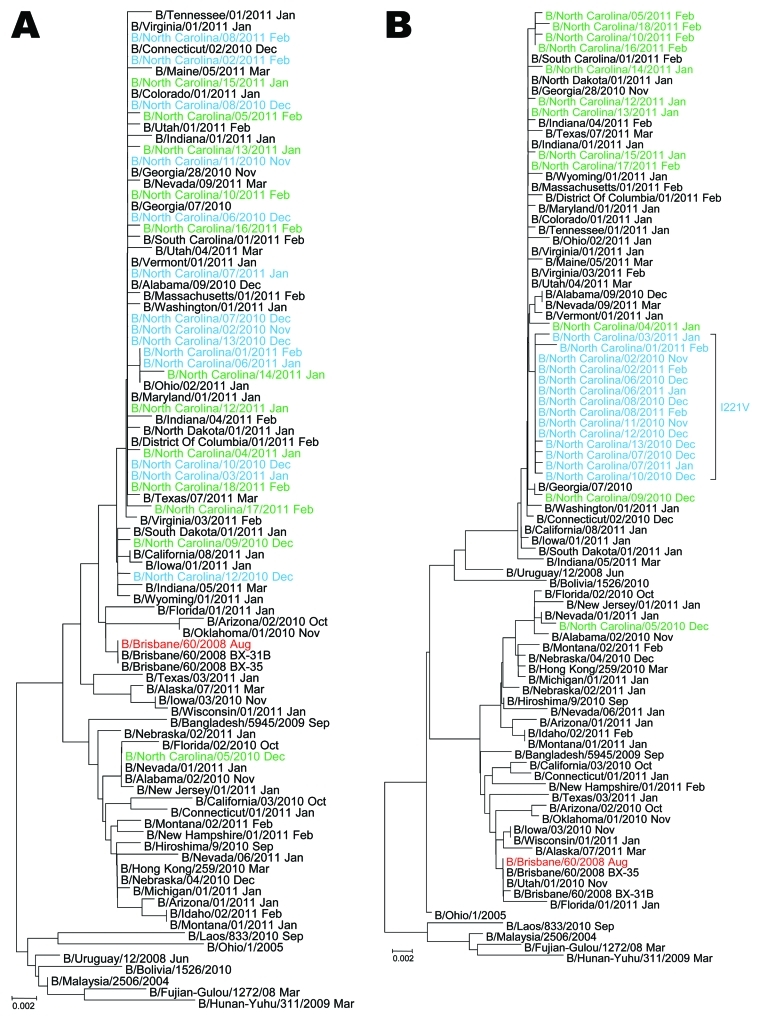
Phylogenetic analysis of A) hemagglutinin and B) neuraminidase genes of Victoria lineage type B influenza viruses (n = 89). Red indicates the 2010−2011 Northern Hemisphere vaccine strain; blue indicates the cluster of influenza B viruses identified in North Carolina carrying the I221V substitution in the neuraminidase; green indicates viruses collected from North Carolina with wild-type sequence at position 221 in the neuraminidase; black indicates representatives of globally circulating influenza B viruses. Month of collection is shown after virus strain designation. Evolutionary distances were computed by using the Tamura-Nei method (www.megasoftware.net/WebHelp/part_iv___evolutionary_analysis/computing_evolutionary_distances/distance_models/nucleotide_substitution_models/hc_tamura_nei_distance.htm). Scale bars indicate number of base substitutions per site.

## References

[1] Monto AS, McKimm-Breschkin JL, Macken C, Hampson AW, Hay A, Klimov A, Detection of influenza viruses resistant to neuraminidase inhibitors in global surveillance during the first 3 years of their use. Antimicrob Agents Chemother. 2006;50:2395–402. 10.1128/AAC.01339-05 16801417 PMC1489772

[2] Carr S, Ilyushina NA, Franks J, Adderson EE, Caniza M, Govorkova EA, Oseltamivir-resistant influenza A and B viruses pre- and post-viral therapy in children and young adults with cancer. Pediatr Infect Dis J. 2011;30:284–8. 10.1097/INF.0b013e3181ff863b 21048522 PMC3070406

[3] Kawai N, Ikematsu H, Iwaki N, Maeda T, Satoh I, Hirotsu N, A comparison of the effectiveness of oseltamivir for the treatment of influenza A and influenza B: a Japanese multicenter study of the 2003–2004 and 2004–2005 influenza seasons. Clin Infect Dis. 2006;43:439–44. 10.1086/505868 16838232

[4] Gubareva LV, Matrosovich MN, Brenner MK, Bethell RC, Webster RG. Evidence for zanamivir resistance in an immunocompromised child infected with influenza B virus. J Infect Dis. 1998;178:1257–62. 10.1086/314440 9780244

[5] Hatakeyama S, Sugaya N, Ito M, Yamazaki M, Ichikawa M, Kimura K, Emergence of influenza B viruses with reduced sensitivity to neuraminidase inhibitors. JAMA. 2007;297:1435–42. 10.1001/jama.297.13.1435 17405969

[6] Sugaya N, Tamura D, Yamazaki M, Ichikawa M, Kawakami C, Kawaoka Y, Comparison of the clinical effectiveness of oseltamivir and zanamivir against influenza infection in children. Clin Infect Dis. 2008;47:339–45. 10.1086/589748 18582202

[7] Hurt AC, Kimm-Breschkin JL, McDonald M, Barr IG, Komadina N, Hampson AW. Identification of a human influenza type B strain with reduced sensitivity to neuraminidase inhibitor drugs. Virus Res. 2004;103:205–11. 10.1016/j.virusres.2004.02.035 15163511

[8] Hurt AC, Iannello P, Jachno K, Komadina N, Hampson AW, Barr IG, Neuraminidase inhibitor-resistant and -sensitive influenza B viruses isolated from an untreated human patient. Antimicrob Agents Chemother. 2006;50:1872–4. 10.1128/AAC.50.5.1872-1874.2006 16641465 PMC1472236

[9] Sheu TG, Deyde VM, Okomo-Adhiambo M, Garten RJ, Xu X, Bright RA, Surveillance for neuraminidase inhibitor resistance among human influenza A and B viruses circulating worldwide from 2004 to 2008. Antimicrob Agents Chemother. 2008;52:3284–92. 10.1128/AAC.00555-08 18625765 PMC2533500

[10] Sheu TG, Deyde VM, Garten RJ, Klimov AI, Gubareva LV. Detection of antiviral resistance and genetic lineage markers in influenza B virus neuraminidase using pyrosequencing. Antiviral Res. 2010;85:354–60. 10.1016/j.antiviral.2009.10.022 19887086

[11] Nguyen HT, Fry AM, Loveless PA, Klimov AI, Gubareva LV. Recovery of a multidrug-resistant strain of pandemic influenza A 2009 (H1N1) virus carrying a dual H275Y/I223R mutation from a child after prolonged treatment with oseltamivir. Clin Infect Dis. 2010;51:983–4. 10.1086/656439 20858074

[12] van der Vries E, Stelma FF, Boucher CAB. Emergence of a multidrug-resistant pandemic influenza A(H1N1) virus. N Engl J Med. 2010;363:1381–2. 10.1056/NEJMc1003749 20879894

[13] Hurt AC, Holien JK, Barr IG. In vitro generation of neuraminidase inhibitor resistance in A(H5N1) influenza viruses. Antimicrob Agents Chemother. 2009;53:4433–40. 10.1128/AAC.00334-09 19651908 PMC2764219

[14] Baz M, Abed Y, McDonald J, Boivin G. Characterization of a multidrug-resistant influenza A/H3N2 virus shed during 1 year by an immunocompromised child. Clin Infect Dis. 2006;43:1555–61. 10.1086/508777 17109288

[15] Okomo-Adhiambo M, Demmler-Harrison GJ, Deyde VM, Sheu TG, Xu X, Klimov AI, Detection of E119V and E119I mutations in influenza A (H3N2) viruses isolated from an immunocompromised patient: challenges in diagnosis of oseltamivir resistance. Antimicrob Agents Chemother. 2010;54:1834–41. 10.1128/AAC.01608-09 20194700 PMC2863645

